# Advances in fiducial‐free image‐guidance for spinal radiosurgery with CyberKnife – a phantom study

**DOI:** 10.1120/jacmp.v12i2.3446

**Published:** 2010-12-22

**Authors:** Christoph Fürweger, Christian Drexler, Markus Kufeld, Alexander Muacevic, Berndt Wowra

**Affiliations:** ^1^ European Cyberknife Center Munich Munich Germany

**Keywords:** image guidance, spinal radiosurgery, fiducial‐free registration, CyberKnife

## Abstract

The image‐guided CyberKnife radiosurgery system is capable of tracking spinal targets without fiducial implants. Recently, a new version of this fiducial‐free image guidance modality (“enhanced Xsight spine tracking”) has been introduced. We assessed the accuracy of this novel technique versus its precursor in a comparative phantom study. The CyberKnife consists of a 6 MV linac on a six‐axis robot and a stereoscopic kV image guidance system. An anthropomorphic head‐and‐neck phantom with a cervical spine section was mounted on the linac nozzle. The robotic manipulator was used to precisely move the phantom to defined positions in the CyberKnife workspace. Multiple stereoscopic images were acquired at different translational and rotational positions. The enhanced Xsight spine tracking readouts were recorded and compared to the nominal phantom position. These tests were repeated with the original Xsight spine tracking version to analyze potential differences. Enhanced Xsight spine tracking correctly reported translational offsets with an RMS error of less than 0.4 mm. Yaw and roll rotations were detected with an accuracy of 0.2°, 0.25°. Pitch offsets were slightly underestimated, with up to 0.3° for an offset of ± 2°. Nominal X (left‐right) translational offsets were partially misinterpreted as roll (0.2° at a 10 mm offset). Apart from this, no correlation between rotational and translational directions was found. In comparison, the original Xsight spine tracking showed identical results for translations, but larger systematic and statistical errors for rotations. Enhanced Xsight spine tracking measurably improves precision in fiducial‐free spinal radiosurgery with the CyberKnife.

PACS number: 87.53.Ly

## I. INTRODUCTION

Spinal radiosurgery requires precise delivery of large radiation doses to lesions close to the spinal cord. Current delivery systems use image guidance techniques such as kV or MV on‐board imaging (OBI), cone beam computed tomography (CT) or stereoscopic imaging to locate the target for patient setup and during treatment.^(1)^ Spinal image guidance relies either on radio‐opaque marker implants^(^ ^2^ ^,^ ^3^ ^)^ or the bony structure of the spine as a noninvasive alternative.^(^ ^4^ ^,^ ^5^ ^,^ ^6^ ^)^ Digitally reconstructed radiographs (DRRs) calculated from the planning CT or a volumetric dataset are used as the reference for patient position and orientation.

From a basic, metrological perspective, all “image guidance” techniques correspond to a measurement of the target position, with the imaging system as the measuring device. As such, the measured target position is subject to a measuring error, consisting of a statistical and, possibly, a systematic component. Sources of error include technical aspects (alignment and calibration of the imaging devices, resolutions limits), robustness of the image matching algorithm, or user variation for systems dependent on manual input. The overall precision of an image‐guided device can only be rated if the characteristics of the image guidance system have been adequately assessed.

The CyberKnife (CK) is a frameless radiosurgery system with a compact 6 MV linac mounted on a robotic arm, which is guided by a stereoscopic imaging system. The patient is positioned on a motorized treatment couch between two amorphous silicon detector panels and ceiling‐mounted diagnostic X‐ray sources. In a typical treatment, more than 100 noncoplanar beams are delivered to the lesion by moving the linac head over a large solid angle around the patient. Since 2005, the CyberKnife is capable of targeting spine lesions by tracking the skeletal structure of adjacent vertebrae. The accuracy of this tracking method has been extensively investigated.^(^ ^7^ ^,^ ^8^ ^)^

At the end of 2009, a major revision of this CK spine tracking feature (“enhanced Xsight spine tracking”) was introduced by the manufacturer (Accuray Inc., Sunnyvale, CA, USA). We quantified measuring errors and analyzed the characteristics of this new modality in comparative phantom tests.

## II. MATERIALS AND METHODS

### A. “Standard” Xsight spine tracking

**Figure 1:**
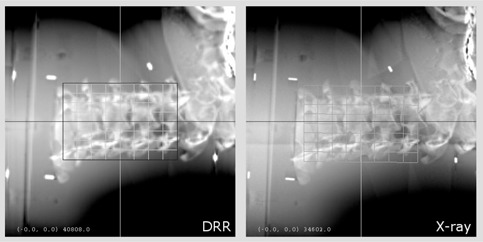
Xsight spine tracking. A cervical spine DRR (left) for one projection is shown together with the corresponding X‐ray live image (right) of the head‐and‐neck phantom. The black rectangle in the DRR denotes the predefined tracking region of interest, which includes only skeletal information of the spine. The matching grid^(9)^ in the X‐ray live image denotes the corresponding region matched by the XST algorithm.

The original Xsight spine tracking (XST) has already been described in detail.^(9)^ Briefly, the XST system compares stereoscopic kV live images of the patient's spine with a pair of DRRs. The DRRs are precalculated from the planning CT dataset in a ray‐casting process with data sampling of 2 mm along the kV X‐ray beam. The spinal segment adjacent to the lesion – typically including approximately three to four adjacent vertebrae – is matched in the reference DRRs and live images (Fig. 1) using a grid‐based, automatic, nonrigid formalism.^(9)^ In this way, the displacement of the target is computed in three translational and three rotational degrees of freedom. This position data is acquired periodically during treatment, and is used to direct the beam to the current position of the target.

### B. Enhanced Xsight spine tracking

Enhanced XST has been modified in several key ways. First, the DRR pair has been replaced by a library of DRRs. The ray‐casting process for DRR calculation uses a reduced sampling step size of 0.5 mm, improving the quality of the reference images. The library consists of 17 DRRs for each stereoscopic imaging plane. Each DRR pair represents a specific patient roll angle (± 5°,± 4°,± 3°,± 2.5°,± 2°,± 1.5°,± 1°,± 0.5°, 0°). Furthermore, automatic, histogram‐based preprocessing of live images has been implemented to improve live‐image characteristics. The patient roll angle is determined by classification of these live images within the DRR library and interpolation, using a matching region of interest similar to the standard version. Translational and pitch/yaw offsets are computed similar to the standard XST, using the matched DRR pair.

### C. Phantom tracking tests

**Figure 2:**
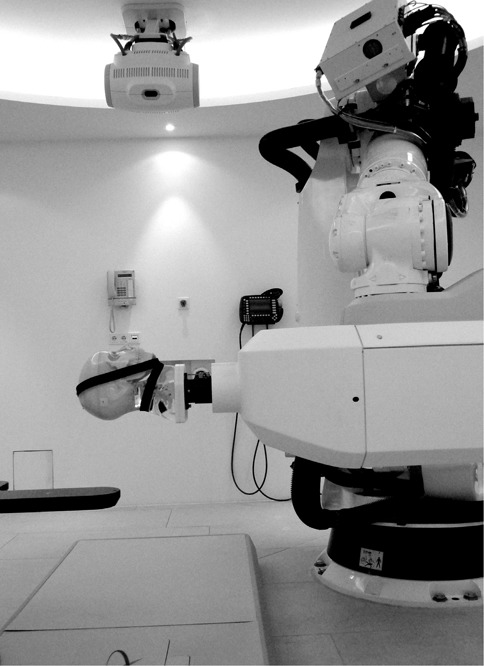
Phantom tracking test setup. The socket of the anthropomorphic head‐and‐neck phantom is rigidly attached to the linac nozzle. Position and orientation presets of the phantom spine are reached by moving the robotic manipulator.

We analyzed the performance of the enhanced XST in tracking tests with the cervical spine of a head‐and‐neck phantom. A CT dataset (1 mm slice thickness, no gap) was acquired to generate phantom treatment plans. DRRs with standard ray‐casting (2 mm step size, standard XST) and a library of improved DRRs (0.5 mm step size, enhanced XST) were calculated. The phantom was firmly mounted on the linac nozzle. The robot (repeatability: ± 0.12 mm) was used as a tool to precisely move the phantom (Fig. 2). Four fiducials implanted into the phantom were used as defined position markers for initial alignment of the phantom. Using the known fiducial locations, the spine region of interest was positioned at the origin of the imaging frame of reference and aligned to its coordinate axes. Offset positions were approached by moving the robot. Translational test positions were ± 10,± 6,± 2 and 0 mm from the reference position. The ranges of rotational offsets were [−5°;+5°] with 0.5° steps for yaw, [−2°;+2°] with 0.25° steps for pitch and [−2°;+2°] with 0.15° steps for roll. The cervical spine section of the phantom was used as tracking target. The tracking region of interest was limited to the vertebral part of the phantom only, with no parts of the skull included (Fig. 1). Image registration was performed automatically by the tracking system with no manual adjustment by the user. The system readings for at least 10 pairs of X‐ray images were recorded in six degrees of freedom at each position. In order to assess a potential performance improvement, we compared the enhanced XST version with the standard XST.

## III. RESULTS

### A. Translations

**Figure 3:**
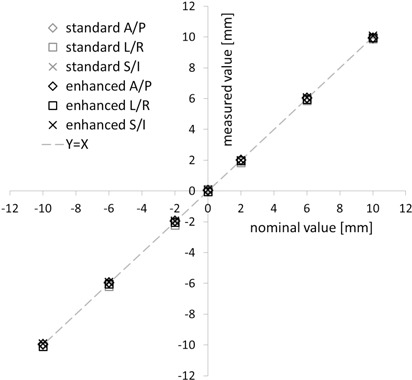
Translational offset detection. Offsets reported by the standard and enhanced spine tracking system (y‐axis) are shown vs. the nominal phantom position (x‐axis) for all three directions – anterior/posterior (A/P), left/right (L/R) and superior/inferior (S/I). Results are means of 10 measurements. Standard deviations (σ ≤ 0.1 mm) are omitted for better readability.

The enhanced XST correctly reported translational offsets in all three directions. Over the whole range (−10 to +10 mm), the mean values deviated from the nominal offset by 0.2 mm or less (Fig. 3), which amounts to an RMS error of less than 0.4 mm. Enhanced XST readings are very reproducible, with a standard deviation σ of less than 0.05 mm for all data points. Considering a robot repeatability of 0.12 mm, the detected deviations are very close or below the limits of our measurement configuration for all data points, indicating a negligible systematic error for translations. This behavior is identical to the standard XST.

### B. Rotations

**Figure 4:**
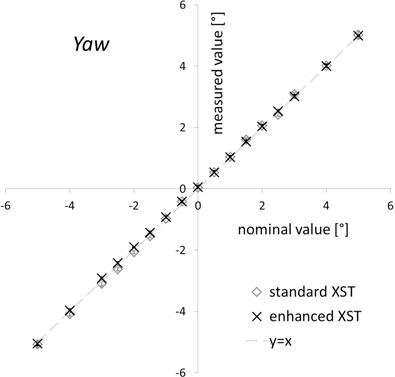
Yaw detection. Yaw offsets reported by standard and enhanced spine tracking (y‐axis) are shown vs. the nominal phantom yaw orientation (y‐axis). Results are means of 10 measurements ± standard deviation.

**Figure 5:**
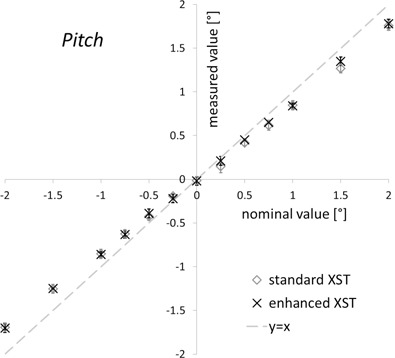
Pitch detection. Pitch offsets reported by standard and enhanced spine tracking (y‐axis) are shown vs. the nominal phantom pitch orientation (y‐axis). Results are means of 10 measurements ± standard deviation.

The accuracy of rotational offset detection by enhanced XST depended on the specific direction. The mean yaw values deviated from the nominal offset by 0.1° or less (Fig. 4) for the complete range of values from −5° to +5°. Standard deviations were smaller than 0.1°. For pitch, offsets were underestimated by up to 15% (or ± 0.15° for a ± 1° offset, Fig. 5), with standard deviations of around 0.05°. Comparison with the standard XST results showed that the characteristics of the mean values were identical within the limits of our measurement technique. The standard deviations for both pitch and yaw were smaller for the enhanced XST, although not by a significant amount (p=0.34 and 0.35, Student's t‐test).

**Figure 6:**
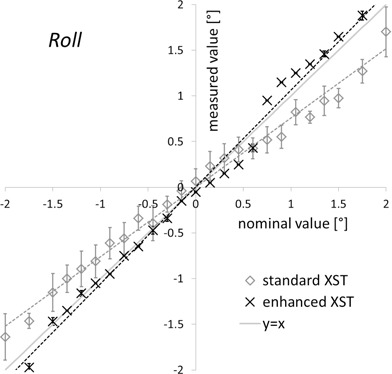
Roll detection. Roll offsets reported by standard and enhanced spine tracking (y‐axis) are shown vs. the nominal phantom roll orientation (y‐axis). Results are means of more than 10 measurements ± standard deviation.

Roll angle results differed greatly between standard and enhanced XST (Fig. 6). On average, the standard version underestimated roll offsets by 25% (e.g., by 0.5° at ± 2°), with a large statistical uncertainty of measured values (σ=± 0.2°). For enhanced XST, the roll estimate is significantly ($p=2.2\times 10^{-4}$, Wilcoxon signed‐rank test) closer to the nominal value, with errors lower than 0.25° over the ± 2° range. Enhanced XST measurements yielded no systematic over‐/underestimation of roll offsets. In addition, the statistical range of the values reported was reduced by more than half ($\sigma <\pm 0.1{}^{\circ},p=2.9\times 10^{-14}$, Student's t‐test).

### C. Cross‐relation errors

**Figure 7:**
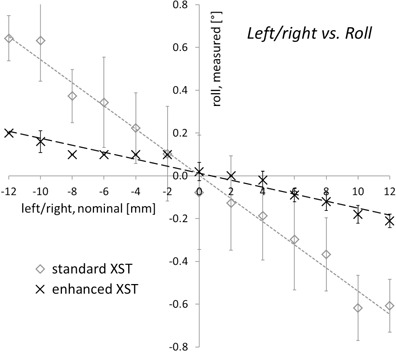
Detected roll offsets as a function of left/right displacements. For a nominal phantom orientation of 0° roll, the roll offsets reported by standard and enhanced spine tracking are given depending on the translational position of the phantom. Results are means of 10 measurements ± standard deviation.

For both XST versions, detection of anterior/posterior and superior/inferior offsets was found to be independent of the phantom pitch and yaw position, and vice versa (data not shown). There was evidence for a partial, systematic misinterpretation of nominal X (left‐right) offsets as roll (Fig. 7), which was significantly less pronounced ($p=2.2\times 10^{-3}$, Wilcoxon signed‐rank test) for the enhanced XST (0.2° at a 10 mm offset) compared to the standard version (0.5° at 10 mm).

## IV. DISCUSSION

The detection of spine rotations was significantly improved for the enhanced XST. Especially for the roll estimate, where the standard XST was subject to a larger statistical and systematic error than pitch and yaw combined, an improvement both in terms of absolute values and statistical range was demonstrated. Rotational errors can be of clinical relevance for specific cases with steep dose gradients to adjacent organs at risk,^(10)^ which are typical for CK spinal treatments. As a consequence, the application of enhanced XST can be expected to induce a clinical benefit in some cases.

We would like to point out that our investigation was limited to measurements with a rigid, nondeformable spine phantom. As a consequence, spine deformation may possibly add an additional error component to the accuracy in patient treatments. However, given the fact that the XST region of interest typically includes only three adjacent vertebrae, the amount of deformation encountered is small, thus limiting the impact on tracking accuracy. Furthermore, preliminary patient tracking data (not given here) shows that the amount of roll fluctuation during treatment is greatly reduced in comparison to the standard XST, which directly reflects the results from the phantom tracking tests. This indicates that even a rigid phantom is a fairly suitable model for a meaningful analysis of XST tracking characteristics.

In current clinical practice, very different image‐guided systems are utilized for stereotactic spinal treatments, ranging from dedicated radiosurgery devices^(^ ^5^ ^,^ ^6^ ^)^ to conventional gantry‐based linacs^(11)^ or tomotherapy.^(12)^ For the enhanced XST modality of the CyberKnife, we have measured a maximum RMS error of 0.4 mm for translational offsets. This compares favorably to the 1.3 mm recently reported for the stereoscopic ExacTrac 6d system,^(5)^ to 1–2 mm for tomotherapy MVCT^(13)^ and to the 0.8 mm for cone beam CT^(5)^ in comparable spine phantom tests. Differences in image guidance precision are significant and will be reflected in delivery. As a consequence, comparative treatment planning studies must include accuracy considerations in order to provide meaningful results.

However, we want to stress that the overall accuracy of a system must not be appraised based on image guidance characteristics only. Even for systems with similar, stereoscopic kV image guidance systems such as CyberKnife and Novalis TX, different interfaces with the corresponding delivery device add system‐specific errors. In an analysis of overall system accuracy, the radiation isocenter of a Novalis TX was reported to deviate from the imaging center by 0.3 mm^(14)^ whereas, for the CyberKnife, the so‐called “machine center” (the equivalence to the gantry “isocenter”) coincides with the imaging center for conceptual reasons.^(15)^ Therefore, it is essential to consider all potential error sources when comparing system accuracy.

## V. CONCLUSIONS

Enhanced Xsight spine tracking further improves CyberKnife image guidance for spinal targets. Submillimeter translational accuracy and a more robust and accurate detection of the spine orientation raises the level of precision achievable in fiducial‐free radiosurgery.
